# Biomarkers of interstitial lung disease associated with primary Sjögren's syndrome

**DOI:** 10.1186/s40001-022-00828-3

**Published:** 2022-10-10

**Authors:** Lin Weng, Yaqiong Chen, Tao Liang, Yihua Lin, Dehao Liu, Ciyong Yu, Yudi Hu, Wei Lui, Yongliang Liu, Xiangfang Chen, Qiyuan Li, Shengxiang Ge, Dana P. Ascherman, Juan Chen

**Affiliations:** 1 Department of Rheumatology, The First Affiliated Hospital of Xiamen University, School of Medicine, Xiamen University, Xiamen, 361003 China; 2 School of Life Sciences, Xiamen University, Xiamen, China; 3 Department of Respiratory Medicine, The First Affiliated Hospital of Xiamen University, School of Medicine, Xiamen University, Xiamen, China; 4 Department of Radiology, The First Affiliated Hospital of Xiamen University, School of Medicine, Xiamen University, Xiamen, China; 5 School of Medicine, Xiamen University, Xiamen, China; 6 State Key Laboratory of Molecular Vaccinology and Molecular Diagnostics, School of Public Health, Xiamen University, Xiamen, China; 7 Fuqing City Hospital affiliated to Fujian Medical University, Fuzhou, China; 8 Department of Pediatrics, School of Medicine, The First Affiliated Hospital of Xiamen University National Institute of Data Science in Health and Medicine, Xiamen University, Xiamen, China; 9 Division of Rheumatology and Clinical Immunology, University of Pittsburgh School of Medicine, Pittsburgh, PA 15261 USA

**Keywords:** Interstitial lung disease (ILD), Serum biomarkers, Predictive factor, Primary Sjögren's syndrome (pSS)

## Abstract

**Objectives:**

The aim of this study was to investigate serum biomarkers linked to primary Sjögren's syndrome (pSS)-associated interstitial lung disease (ILD).

**Methods:**

69 pSS patients were consecutively enrolled and evaluated via quantitative ILD scoring based on high-resolution computed tomography (HRCT). Biomarkers of interest were assessed by multiplex enzyme-linked immunosorbent assays (ELISAs).

**Results:**

Among consecutively enrolled patients with pSS, the presence of pSS–ILD was 50% based on the presence of radiographically defined interstitial lung abnormalities (ILA) meeting specified criteria for mild/moderate (ILA 2) or severe (ILA 3) disease. Age, immunoglobulin M (IgM), C-reactive protein (CRP), and serum levels of eotaxin/CCL11, Krebs von den Lungen-6 (KL-6), TNFα, and TGFα were significantly higher in the combined pSS–ILD group (ILA 2 + ILA 3) than in the pSS–no-ILD and pSS–indeterminate ILD groups (ILA 0 and ILA 1, respectively) in unadjusted analyses (*p* < 0.05 for all variables). A binary logistic regression model revealed that disease duration and KL-6 levels were associated with the presence of pSS–ILD (*p* < 0.05). Complementary least absolute shrinkage and selection operator (LASSO) modeling showed that age, KL-6, and TNF-α effectively differentiated pSS–ILD (ILA 2 + ILA3) from pSS without ILD (ILA 0 + ILA 1), with an area under the curve (AUC) of 0.883 (*p* value < 0.0001).

**Conclusions:**

Patient age, disease duration, and serum levels of both KL-6 and TNFα were the most discriminating factors associated with the presence of ILD in our pSS patients. Higher levels of CRP, IgM, eotaxin, TGFα, and TNFα should also prompt the search for occult as well as clinically evident lung involvement based on statistically significant univariate associations with pSS–ILD.

**Clinical trial registration:**

None.

**Supplementary Information:**

The online version contains supplementary material available at 10.1186/s40001-022-00828-3.

## Background

Interstitial lung diseases (ILD) consist of a heterogeneous group of parenchymal lung disorders that are characterized by variable degrees of inflammation and fibrosis [1, 2]. Although different subsets of ILD may share common radiologic, pathologic, and clinical manifestations, they are associated with quite different etiologies and co-morbidities [3]. In primary Sjögren's syndrome (pSS), the annual incidence of respiratory manifestations is estimated at 10% 1 year after diagnosis and increases to 20% after 5 years [4]. In a recent report from China, the prevalence of ILD in pSS among those undergoing HRCT examination is as high as 39.1% [5]. Importantly, patients with pSS and lung involvement have an increased risk of death in comparison with those without lung involvement [6], with an estimated 5-year survival rate ranging from 84% [7] to 87.3% [8].

While 10–51% of patients develop ILD years before the onset of pSS [9], pSS–ILD begins at the same time as other systemic manifestations in approximately 10% of cases. In the remainder of cases, ILD develops late in the course of disease [10]. High resolution computed tomography (HRCT) of the chest represents the main imaging tool for evaluating pSS-related pulmonary abnormalities, because HRCT is very sensitive in detecting mild pSS-related pulmonary abnormalities, even in asymptomatic patients [10]. However, screening pSS patients with HRCT is not currently standard of care, despite the relatively high prevalence of ILD in this disease and the potential for significant morbidity. Therefore, identifying clinical risk factors and other serum protein biomarkers associated with the development of pSS–ILD will be critically important in facilitating non-invasive detection of lung involvement at earlier, more treatable stages of disease.

Previous studies have shown that prognostic factors associated with the occurrence of pSS–ILD include older age, male sex, disease duration, smoking, an increase in anti-nuclear antibodies or rheumatoid factor, the presence of anti-SSA/Ro52 antibodies [11], low levels of circulating C3, and increased C-reactive protein levels [12–14]. However, there are few reports on other serum protein biomarkers in pSS–ILD that potentially shed light on underlying disease mechanisms. In this study, we, therefore, focused on clinical risk factors and alternative serum protein biomarkers capable of discriminating pSS–ILD from pSS–no-ILD.

## Materials and methods

### Patients

Sixty-nine patients with pSS who met the 2016 American College of Rheumatology (ACR)–European League Against Rheumatism (EULAR) classification criteria for primary Sjögren's syndrome [15, 16] were consecutively enrolled between September 2013 and June 2017 through the Department of Rheumatology at the first affiliated Hospital of Xiamen University, School of Medicine, Xiamen University and Fuqing City Hospital affiliated with Fujian Medical University, China. Recruited patients in this observational study were from different provinces, mainly in the south of China. Patients with Sjogren’s features occurring in the context of other well-defined connective tissue diseases (CTD) such as rheumatoid arthritis, systemic lupus erythematosus, mixed connective tissue disease, polymyositis, and dermatomyositis were excluded. Other illnesses producing clinical manifestations of xerophthalmia and/or xerostomia—such as past history of head and neck irradiation, pre-existing lymphoma, hepatitis B or C infection, acquired immunodeficiency, sarcoidosis, or graft vs. host disease—were also excluded. For eligible participants, patient-specific variables, including age, sex, and disease duration, were recorded. HRCT images were obtained from all patients at enrollment (see below for details). Serum samples were obtained at the time of clinical data collection for all of the consecutively enrolled patients and stored at – 80 ℃.

Ethics approval for the study was obtained from the Ethics Committee of the first affiliated Hospital of Xiamen University, School of Medicine, Xiamen University, China (Approval number: KY2017-026). Written informed consent was obtained from each participant before enrollment.

### Laboratory testing

Autoimmune serology, serum immunoglobulin levels (including IgG, IgA and IgM), C3, C4, and C-reactive protein (CRP) were assessed in all patients. Antinuclear antibody (ANA) testing was performed using indirect immunofluorescence and qualitatively graded based on the serum dilution yielding positive staining (ANA+ = ANA 1:80 − 1:160; ANA++ = ANA 1:320 − 1:640; ANA+++ = ANA ≥ 1:1280). Anti-SSA/Ro60, anti-Ro52, and anti-SSB/La antibodies were assessed via immunoblotting.

### HRCT and ILD scoring

HRCT (Aquilion 16; Toshiba Medical Systems) of the chest without contrast was performed for all patients during end inspiration using 1–2-mm collimation at 1–2-mm intervals in the supine position. HRCT classification was based on readings from at least two independent pulmonologists/radiologists blinded to the disease characteristics of the patients. Although the intra- and inter-observer correlations exceeded 90% for the HRCT image readers in previous studies, discrepancies in the current study were resolved by consensus agreement with one additional reader. Radiographic features indicative of ILD included ground-glass opacities, septal thickening, reticulation, traction bronchiectasis, and/or honeycombing. Additional characterization utilized a scale of interstitial lung abnormalities (ILAs) ranging from 0 to 3 [17], where ILA 0 = no ILD, ILA 1 = indeterminate ILD (focal or unilateral ground-glass attenuation, focal or unilateral reticulation, or patchy ground-glass abnormality involving < 5% of the lung), ILA 2 = mild/moderate ILD (changes affecting > 5% of any lobar region with non-dependent ground-glass or reticular abnormalities, diffuse centrilobular nodularity, non-emphysematous cysts, honeycombing, or traction bronchiectasis), ILA 3 = advanced ILD (bilateral fibrosis in multiple lobes associated with honeycombing and traction bronchiectasis in a subpleural distribution) [18, 19]. In this study, ILA scores ≥ 2 identified patients with pSS–ILD, while ILA scores of 0 and 1 encompassed pSS patients without definite ILD.

### Detection of pSS–ILD-related serum biomarkers by multiplex ELISA

Multiplex enzyme-linked immunosorbent assays (Multiplex ELISAs) were performed using Luminex xMAP technology according to the manufacturer’s instructions (EMD Millipore Corporation, Billerica, MA, USA) [20]. A combined 42-plex assay was used to determine serum levels of a range of cytokines, chemokines and MMPs (listed in Additional file 1) potentially related to the mechanism of pSS–ILD.

### Detection of KL-6 by CLEIA

The concentration of Krebs von den Lungen-6 (KL-6) was measured using a commercial chemiluminescent enzyme immunoassay (CLEIA) according to the experimental procedure specified by the manufacturer (Fujirebio Inc., Tokyo, Japan).

### Statistical analysis

All continuous variables were evaluated for normality to ensure uniformity in concentration and dispersion. Median and interquartile range (IQR) were presented for variables that did not follow a normal distribution.

Bivariate analyses were conducted using Chi-square or Fisher’s exact test for categorical variables and outcomes (pSS–ILD and pSS no/indeterminate-ILD). Alternatively, Mann–Whitney *U* tests were used for non-parametric continuous variables (e.g., serum biomarker levels, risk scores) and outcomes. To determine correlations among continuous variables and identify potential confounding factors, Spearman correlation coefficients were calculated.

Additional analyses were conducted to determine which demographic variables, clinical risk factors, and/or serum proteins were associated with pSS–ILD. These variables were included in the final logistic regression model as candidate covariates to assess associations with pSS–ILD. Risk scores were derived for each patient based on the final logistic regression model. We further evaluated the performance of our model using receiver operating characteristic (ROC) analysis to calculate area under the curve (AUC) values.

Finally, least absolute shrinkage and selection operator (LASSO) modeling was used as a penalized regression tool to develop a clinical prediction algorithm for detecting the presence of pSS–ILD. We determined the lowest shrinkage parameter (λ) with which to select final clinical risk factors and protein biomarkers (and their coefficients) for predicting the probability of being diagnosed as having pSS–ILD. ROC curves were then generated to assess the ability of algorithm-based predictions to discriminate between the presence vs. absence of pSS–ILD (as measured by AUC).

All statistical analyses were performed using IBM SPSS Statistics, version 20.0. Two-sided *p* values ≤ 0.05 were considered statistically significant. To adjust for multiple comparisons, *p* values were corrected by the false discovery rate (FDR).

## Results

### Patient-specific variables and pSS–ILD

Demographic characteristics of our pSS cohort are summarized in Table 1. Most patients were female (84%, n = 58), and the mean age at the time of diagnosis of pSS was 55.04 ± 12.90 years (Table 1). Among the 69 patients with pSS, 19 (28%) had advanced pSS–ILD (ILA score 3), 15 (22%) had mild/moderate pSS–ILD (ILA score 2), 25 (36%) had indeterminate pSS–ILD (ILA score 1), and 10 (14%) had pSS–no ILD (ILA score 0) based on HRCT criteria (Table 1). After combining individuals with ILA score 2 + 3, the prevalence of pSS–ILD in our cohort was 50%.

**Table 1:** Clinical, demographic, and laboratory characteristics of pSS patients with different stages of ILD

	pSS-no ILD	pSS-indeterminate ILD		pSS-mild/moderate ILD		pSS-advanced ILD	
	(ILA score 0)	(ILA score 1)		(ILA score 2)		(ILA score 3)	
Demographic parameters							
Number, no. (%)	10 (14)		25 (36)		15 (22)		19 (28)
Female, no. (%)	9 (90)		21 (84)		13 (87)		15 (79)
Male, no. (%)	1 (10)		4 (16)		2 (13)		4 (21)
Age at diagnosis of pSS, median (IQR), years	49.00 (33.25–53.25)		53.00 (46.50–61.50)		60.00 (48.00–64.00)*		62.00 (55.00–70.00)***
Disease duration, median (IQR), years	1.00 (0.25–5.00)		1.00 (0.75–4.00)		3.00 (1.00–10.00)		2.00 (0.50–5.00)
Laboratory findings							
IgG, g/l	16.85 (13.75–19.23)		18.30 (14.25–21.50)		21.20 (13.50–26.10)		14.00 (10.04–19.20)
IgM, g/l	1.16 (0.77–1.47)		1.00 (0.62–2.39)		1.37 (1.06–2.44)		1.58 (1.38–2.98)*
IgA, g/l	2.75 (2.19–3.92)		3.36 (2.67–4.60)		2.88 (1.67–4.50)		2.60 (2.49–3.82)
C3, g/l	1.01 (0.80–1.25)		0.86 (0.67–1.08)		1.02 (0.98–1.11)		0.93 (0.92–1.12)
C4, g/l	0.22 (0.17–0.31)		0.18 (0.11–0.22)		0.15 (0.12–0.22)		0.28 (0.20–0.31)
CRP, mg/l	0.66 (0.33–5.45)		2.20 (0.40–6.76)		4.9 (1.41–30.00)		9.95 (4.80–22.60)***
ANA positive, no. (%)	7 (70)		20 (80)		12 (80)		10 (50)
Anti-SSA/Ro60 positive, no. (%)	6 (60)		14 (56)		8 (53)		4 (20)**
Anti-SSB/La positive, no. (%)	3 (30)		10 (40)		3 (20)		3 (15)
Anti-Ro-52 positive, no. (%)	7 (70)		16 (64)		9 (60)		12 (60)

*pSS* primary Sjogren’s syndrome, *ILA* interstitial lung abnormalities, *IQR* interquartile range, *ANA* Antinuclear antibody, *IgA* IgM, *IgG* Immunoglobulin AM and G, *C3 and C4* complement 3 and complement 4, *CRP* C-reactive protein. Mann–Whitney *U* test and Fisher’s exact test were used to determine *p* values

^*^p < 0.05, **p < 0.01, ***p < 0.001 when comparing different subcategories of pSS-ILD with pSS-no ILD

Age was significantly different between mild/moderate ILD (ILA score 2) and no-ILD (ILA score 0) (*p* < 0.05), and between advanced ILD (ILA score 3) and pSS no-ILD (ILA score 0) (*p* < 0.001) (Table 1). Overall, the mean age at diagnosis in the combined pSS–ILD group (ILA score 2 + 3) was significantly higher than in individuals who did not meet criteria for pSS–ILD (ILA score 0 + 1) (61 vs. 52, *p* = 0.003) (Table 2). Area under the curve (AUC) derived from ROC analysis was 0.706 (95% confidence interval: 0.582–0.830, *p* = 0.003) for age of pSS diagnosis as a discriminating factor for pSS–ILD (Table 3). In contrast to age at diagnosis, there were no statistically significant differences in gender or disease duration between those meeting criteria for pSS–ILD and those who did not (*p* > 0.05) (Table 2).

**Table 2:** Clinical, demographic, and laboratory characteristics of pSS patients segregated by ILD subcategory

Variable	no/indeterminate ILD	moderate/advanced ILD	*p* value
	(ILA score 0 + 1)	(ILA score 2 + 3)	
	(n = 35)	(n = 34)	
Female, no. (%)	30 (86)	28 (82)	0.703
Age at diagnosis of pSS, years	52.00 (44.00–58.00)	61.00 (52.50–66.50)	**0.003**
Disease duration, years	1.00 (0.50–4.00)	2.50 (1.00–5.00)	0.229
IgG, g/l	17.60 (14.10–21.50)	18.50 (10.09–21.58)	0.792
IgM, g/l	1.09 (0.62–1.86)	1.44 (1.19–2.88)	**0.029**
IgA, g/l	3.21 (2.53–4.37)	2.74 (2.26–3.83)	0.203
C3, g/l	0.89 (0.72–1.19)	0.98 (0.92–1.11)	0.138
C4, g/l	0.20 (0.14–0.24)	0.21 (0.15–0.30)	0.201
CRP, mg/l	2.09 (0.40–6.23)	7.97 (1.85–23.20)	**0.002**
ANA positive, no. (%)	31 (89)	28 (82)	0.695
Anti-SSA/Ro60 positive, no. (%)	23 (66)	15 (44)	0.071
Anti-SSB/La positive, no. (%)	13 (37)	6 (18)	0.07
Anti-Ro-52 positive, no. (%)	26 (74)	28 (82)	0.417
eotaxin/CCL11, pg/ml	278.00 (167.00–391.00)	1130.00 (549.25–1538.25)	**0.005**
KL-6, U/ml	146.00 (119.00–208.00)	202.00 (164.25–300.75)	** < 0.0001**
TGF-α, pg/ml	1.07 (1.07–1.07)^1^	1.07 (1.07–4.19)	**0.044**
TNF-α, pg/ml	7.17 (1.83–12.07)	11.60 (5.88–26.86)	**0.026**

*Eotaxin/CCL11* C–C motif chemokine ligand 11, *KL-6* Krebs vonden Lungen-6, *TGFa* growth factor alpha, *TNFa* tumor necrosis factor-alpha

The continuous variables were presented as median (interquartile range). The significance of differences in demographic variables, clinical features, and serum biomarkers were determined by univariate analyses using Fisher’s exact test for categorical variables and Mann–Whitney *U* test for continuous variables. *IQR* interquartile range

^*^p < 0.05, **p < 0.01, ***p < 0.001 when the pSS moderate/advanced-ILD group (ILA score 2 + 3) was compared with the pSS no/indeterminate-ILD group (ILA score 0 + 1)

^1.^The lower limit of detection for TGF-α was 1.07 pg/ml

Bold values represent the statistic significances p < 0.05

**Table 3:** Factors discriminating pSS–moderate/advanced ILD from pSS–no/indeterminate ILD

Variable	Mild/moderate and severe ILD Disease (ILA score 2 + 3)			
	AUC	95% CI		*p* value
Age at diagnosis of pSS	0.706	0.582–0.830		0.003**
Disease duration	0.584	0.448–0.719		0.232
IgG	0.518	0.377–0.660		0.792
IgM	0.653	0.521–0.785		0.029*
IgA	0.589	0.453–0.725		0.203
C3	0.604	0.467–0.741		0.138
C4	0.590	0.453–0.726		0.201
CRP	0.713	0.590–0.836		0.002**
ANA positive	0.531	0.394–0.668		0.657
Anti-SSA/RO-60 positive	0.608	0.474–0.742		0.123
Anti-SSB/La positive	0.597	0.463–0.732		0.164
Anti-Ro-52 positive	0.540	0.404–0.677		0.565
eotaxin/CCL11	0.695	0.571–0.818		0.006**
KL-6	0.883	0.798–0.969		< 0.0001***
TGF-α	0.618	0.484–0.753		0.091
TNF-a	0.656	0.527–0.785		0.026*

*AUC* area under the curve

^*^p < 0.05, **p < 0.01, ***p < 0.001 when the pSS moderate/advanced-ILD group (ILA score 2 + 3) was compared with the pSS no/indeterminate-ILD group (ILA score 0 + 1)

Bold values represent the statistic significances p < 0.05

**Table 4:** Relationship between selected risk factors and pSS-ILD

	Beta coefficient	*p* value
Disease duration	0.150	0.032
KL-6	0.006	0.000
Constant	− 3.824	0.000

Relationship between selected risk factors and pSS–ILD

Descriptive analyses showed differences in risk of pSS–ILD for seventeen indices mentioned above. We conducted Spearman correlation analyses for continuous variables and selected one member of each pair of correlated variables (r > 0.3 and p < 0.05) to include into the logistic regression model to avoid multicollinearity. With those steps, disease duration and KL-6 were selected to include in the final model.

### Laboratory findings

As shown in Table 1, IgM was significantly different between advanced ILD (ILA score 3) and no-ILD (ILA score 0) in pSS patients (*p* < 0.05). The combined pSS–ILD group (ILA 2 + ILA 3) had higher circulating levels of IgM than the group consisting of pSS–indeterminate and pSS–no ILD (*p* = 0.029) (Table 2), yielding an AUC of 0.653 (95% confidence interval: 0.521–0.785, *p* = 0.029) (Table 3).

CRP was also significantly different between severe ILD and no-ILD in pSS patients (*p* < 0.001) (Table 1). Furthermore, higher levels of CRP distinguished the combined pSS–ILD group (ILA score 2 + 3) from those without definite ILD (*p* = 0.002) (Table 2), yielding an AUC of 0.713 (95% confidence interval: 0.590–0.836, *p* = 0.002 (Table 3). Conversely, there were no statistically significant differences in circulating levels of IgG, IgA, C3, or C4 between the two groups (Table 2).

### Autoantibodies

The percentage of pSS patients with anti-SSA/Ro60 antibodies was significantly higher in the pSS-advanced ILD patients (ILA score 3) than in the pSS no-ILD subgroup (ILA score 0) (*p* < 0.01) (Table 1). However, the percentages of anti-Ro52, anti-SSB/La, and ANA antibody positivity were not significantly different between different pSS subgroups (*p* > 0.05) (Table 2).

### Serum protein biomarkers

To address whether serum protein biomarkers are associated with the presence of pSS–ILD, we measured the serum levels of KL-6 as well as 42 proteins that encompass cytokines, chemokines, growth factors, and remodeling proteins (MMPs) previously examined in IPF and other cohorts of CTD–ILD. We then assessed the relationship between the serum levels of these biomarkers and pSS–ILD disease severity based on HRCT. This analysis revealed that eotaxin/CCL11, KL-6, TGFα and TNFα showed significant associations with ILD severity in selected subgroups of pSS–ILD (Tables 2, 3), whereas no statistically significant differences were found for the other biomarkers.

### Identification of clinical and serum protein biomarkers associated with pSS–ILD

**Fig. 1:**
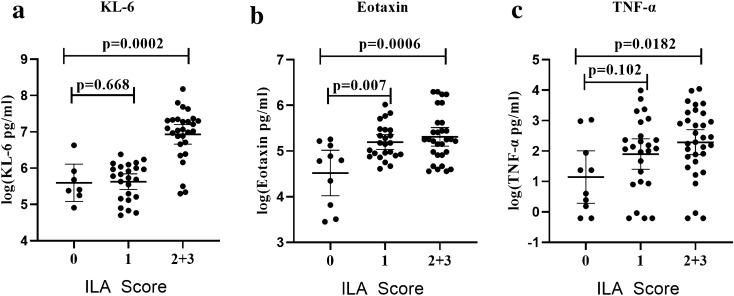
Relationship between serum levels of cytokines and the severity of ILD (ILA score 0 vs. ILA score 2 + 3) by HRCT in pSS. Panels a–c demonstrate the relationship between the natural log of serum a) KL-6, **b** eotaxin/CCL11, and **c** TNFα levels and ILD severity (*p* = 0.0002, 0006, and 0.0182, respectively)**.** Each symbol represents an individual patient; horizontal lines show the mean value (natural log) of serum levels for specified cytokines. *P* values were determined by Mann–Whitney *U* test

Significant differences were found between the pSS moderate/advanced-ILD group (ILA score 2 + 3) and the pSS no-/indeterminate-ILD group (ILA score 0 + 1) based on age, IgM, CRP, eotaxin, KL-6, TGFα and TNFα levels (*p* < 0.05, Table 2 and Fig. 1). Other variables did not show statistically significant differences in univariate analyses. Corrected by FDR, age, CRP, eotaxin, and KL-6 remained statistically significant (*p* = 0.017, 0.017, 0.021, < 0.0001, respectively) (Table 2). In ROC analyses, serum levels of eotaxin, KL-6 and TNFα distinguished combined subgroups of pSS–ILD (ILA score 2 + 3) from subgroups without definite ILD, with respective AUCs of 0.695 (95% confidence interval: 0.571–0.818, *p* = 0.006), 0.883 (95% confidence interval: 0.798–0.969, *p* < 0.0001), and 0.656 (95% confidence interval: 0.527–0.785, *p* = 0.086) (Table 3).

We next used the Spearman coefficient to assess correlations between continuous variables, and then selected only one member of each pair of correlated variables (r > 0.3, *p* < 0.05) to avoid multicollinearity in prediction models. Based on these criteria, disease duration and KL-6 levels were the two variables retained in the final logistic regression model to assess their association with the occurrence of pSS–ILD (*p* < 0.0001, Table 4).

**Fig. 2:**
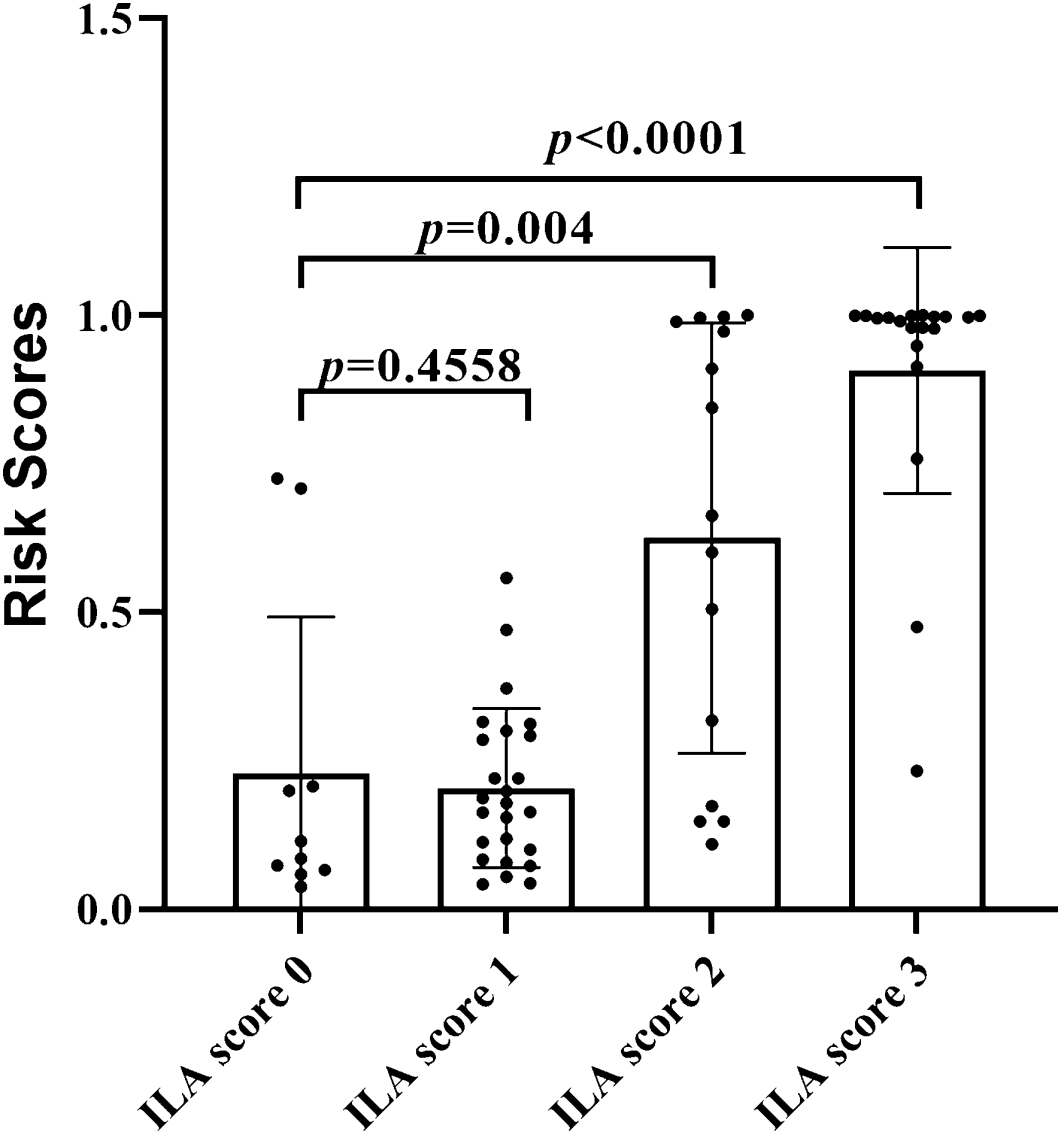
Distribution of risk scores between different ILA subcategories. KL-6 and disease duration were fit into a logistic regression model, yielding an equation for calculation of a combined risk score = − 3.824 + 0.006*KL-6 + 0.150*disease duration. *P* values for ILA score 0 vs. ILA score 1, ILA score 0 vs. ILA score 2 and ILA score 0 vs. ILA score 3 were 0.4558, 0.004 and < 0.0001, respectively. The presented *p* values were determined by the non-parametric Mann–Whitney *U* test

After KL-6 and disease duration were fit into this logistic regression model, we calculated risk scores based on the equation: risk for pSS–ILD = 3.824 + 0.006*KL-6 + 0.150*disease duration. This analysis demonstrated that risk scores were significantly different between different ILA subcategories, as *p* values for comparisons of ILA score 0 vs. ILA score 1, ILA score 0 vs. ILA score 2 and ILA score 0 vs. ILA score 3 were 0.4558, 0.004 and < 0.0001, respectively (Fig. 2). Overall, these data indicated that the model incorporating disease duration and KL-6 levels effectively distinguished pSS–ILD from pSS no-ILD.

### Clinical prediction model

**Fig. 3:**
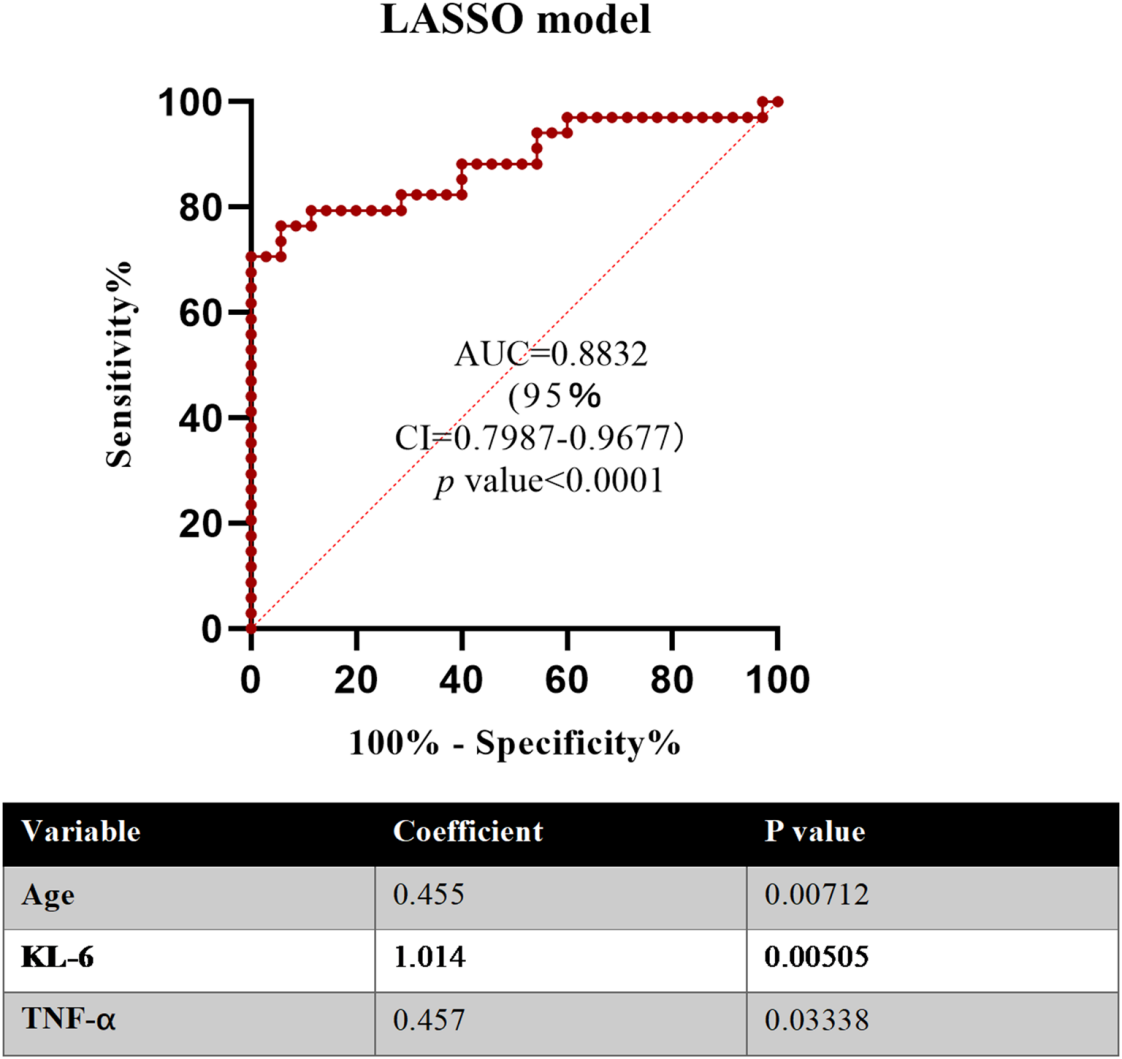
Least absolute shrinkage and selection operator (LASSO) modeling in the identification of pSS–ILD. Application of LASSO regression modeling that is based on machine learning revealed clinical risk factors and serum protein biomarkers capable of distinguishing pSS–ILD patients with moderate/advanced-ILD from those with no-/indeterminate-ILD. The corresponding ROC curve reflects performance characteristics of this model, as indicated by area under the curve (AUC). The accompanying table shows regression coefficients for clinical risk factors and specific serum proteins

To develop complementary clinical prediction tools capable of distinguishing pSS–ILD from pSS–no ILD, we applied LASSO modeling—a machine learning-based, penalized regression method designed to minimize data complexity and maximize precision. This analysis demonstrated that the clinical and serum biomarker signature consisting of age, KL-6, and TNFα effectively differentiated pSS–ILD from pSS–no ILD with high sensitivity and specificity, yielding an AUC of 0.883 (95% CI = 0.7987–0.9677, *p* value < 0.0001) (Fig. 3).

## Discussion

Based on HRCT criteria, the prevalence of pSS–ILD (ILA 2 + ILA 3) in this cohort of consecutively enrolled pSS patents from our center was 50%. Because patients were enrolled consecutively without respect to underlying lung disease, this statistic likely reflects the true frequency of this complication that may go undetected in unscreened populations (note that in another study of 527 patients with pSS, 206 (39.1%) had evidence of ILD [5]). Subgroup comparisons indicated that the occurrence of pSS–ILD was associated with older age and higher serum levels of CRP, immunoglobulin M, eotaxin, KL-6, TGFα and TNFα. Our final binary logistic regression model revealed that KL-6 level and disease duration were key parameters associated with pulmonary involvement in Sjögren’s syndrome patients. LASSO modeling complemented these findings, demonstrating that age, KL-6, and TNFα effectively differentiated pSS–ILD from pSS–no ILD with high sensitivity and specificity.

Advancing age was also strongly associated with the development of pSS–ILD in other studies. A recent systematic review of 6157 pSS patients showed that older age, male sex, and higher CRP levels were risk factors for pSS–ILD [13, 14]. In addition, late age of onset and long duration of disease have also been linked to pSS–ILD [21], consistent with our logistic regression model showing that disease duration of pSS plays a critical role in the development of pSS–ILD.

Regarding relationships between the presence of autoantibodies and the occurrence of pSS–ILD, the percentage of anti-SSA/Ro60 antibody in pSS-advanced ILD patients (ILA score 3) was significantly higher than in pSS–no ILD patients in our study as well as in others [21]. Other studies have also shown that anti-Ro52/La antibodies are an independent risk factor associated with the occurrence of ILD in pSS [11, 21, 22]. Anti-La/SSB positivity as well as high levels of IgG and IgA were shown in a separate study to be independently associated with lung involvement in pSS [23]. Unlike these studies, however, we did not find statistically significant associations between anti-Ro52/La or anti-La/SSB antibodies and pSS–ILD.

Serum levels of eotaxin were higher in the pSS–ILD subgroup of our cohort, distinguishing these pSS patients from those without definite ILD. Eotaxin may be involved in the pathogenesis of ILD in pSS—possibly due to activation of fibroblasts, eosinophils, and neutrophils, each of which bears receptors for this chemokine. Although there have been no reports on eotaxin in the context of pSS–ILD, other studies have shown that eotaxin directly affects lung fibroblasts by upregulating procollagen type I gene expression and collagen protein production in lung fibroblasts—suggesting a potential contribution of this CC chemokine to deposition of extracellular matrix (ECM) and the early phase of tissue remodeling in the lung [24]. Interestingly, a recent RA-ILD study found significant associations between eotaxin and the severity of RA-ILD [25], again consistent with a role for this chemokine in lung fibrosis [24–26].

The present study showed that the serum level of KL-6 was the most discriminating biomarker for pSS–ILD in our cohort. Indeed, levels of KL-6 significantly correlated with the severity of ILD measured by HRCT to a greater degree than other biomarkers associated with pSS–ILD in this study (eotaxin, TGFα and TNFα), as shown in Fig. 1. KL-6 has been classified as a human mucin-like glycoprotein (in the MUC1 family) secreted predominantly by type II pneumocytes in the affected lungs of patients with ILD [27, 28]. Although previous studies have investigated the relationship between serum KL-6 levels and disease activity/severity as well as prognosis in SSc–ILD [29–31], RA-ILD [30, 32, 33], and PM/DM-ILD [30, 32], we found that KL-6 also related to severity of pSS–ILD. Consistent with our findings, Kamiya et al. showed that pSS–ILD patients with higher levels of serum KL-6 (> 800U/mL) had a higher pulmonary mortality rate compared to those without elevated serum KL-6 levels [34]. Overall, the ability of serum levels of KL-6 to distinguish CTD–ILD from CTD–no ILD in multiple subsets of early as well as established disease groups demonstrates its value as a biomarker for these disorders. Whether KL-6 is simply serving as a marker of epithelial cell damage or is playing a direct role in aberrant signaling pathways remains unclear, however.

Our study does have several limitations. First, even though we enrolled patients from different provinces and different hospitals of China, there were small numbers of patients in each subgroup of pSS–ILD, limiting study power. Second, pulmonary function was only tested in 20 patients in this study (data not shown) and, therefore, could not be incorporated into any of our analyses. Of note, however, our findings support the associations between pSS–ILD and serum protein biomarkers, such as eotaxin, KL-6, TGFα, and TNFα. While future cross-sectional studies in larger cohorts will be critical in validating findings from this study, prospective studies will also be important to investigate a wider range of biomarkers and their ability to correlate with disease activity over time. In turn, these corroborative studies will provide greater insight to the underlying pathogenesis of pSS–ILD and elucidate therapeutically targetable pathways.

## Conclusions

Patient age, disease duration, and serum levels of both KL-6 and TNFα were the most discriminating factors associated with the presence of ILD in our cohort of pSS patients. In addition to these parameters, higher levels of CRP, IgM, eotaxin, TGFα, and TNFα should also prompt the search for occult as well as clinically evident lung involvement based on statistically significant univariate associations with pSS–ILD in this study.

## Supplementary Information

**Additional file 1: Table**. CTD–ILD-related biomarkers assessed by Multiplex ELISA.

## Data Availability

The outputs are available from the authors upon request.

## Abbreviations

pSS: Sjögren's syndrome
ILD: Interstitial lung disease
HRCT: High-resolution computed tomography
ILA: Interstitial lung abnormalities
CTD: Connective tissue diseases
CRP: C-reactive protein
ANA: Antinuclear antibody
KL-6: Krebs von den Lungen-6
ROC: Receiver operating characteristic
AUC: Area under the curve
FDR: False discovery rate
LASSO: Least absolute shrinkage and selection operator modeling
