# Supplementary material for: Biomarkers of interstitial lung disease associated with primary Sjögren's syndrome
Source: Eur J Med Res. 2022 Oct 10;27:199. doi: 10.1186/s40001-022-00828-3 (PMC9549683; doi:10.1186/s40001-022-00828-3)
Supplement: Supplementary file 1 — Additional file 1: Table. CTD–ILD-related biomarkers assessed by Multiplex ELISA. [file 40001_2022_828_MOESM1_ESM.docx]

**Table Supplementary.** CTD-ILD related biomarkers assessed by Multiplex ELISA

| Cytokines | IL-1a, IL-1RA, IL-1b, IL-2, IL-4, IL-6, IL-7, IL-8(CXCL8),IL-9 IL-10, IL-11, IL-12(p40), IL-12(p70) IL-15, IL-17, IL-29, TNF-a, TNF-b,  IFNa,IFNg, Flt-3L |
| --- | --- |
| Chemokines | GCP-2(CXCL6),MIG/CXCL9,IP-10CXCL10,I-TAC/CXCL11,Eotaxin/CCL11, MIP-1a、MIP-1b、MIP、MCP-1、MCP-3、Fractalkine |
| Growth Factors | GM-CSF, TGFa, VEGF |
| Remodeling Proteins | MMP-1、MMP-2、MMP-7、MMP-9、MMP-10 |
